# Analysis of Bacterial Diversity in Different Heavy Oil Wells of a Reservoir in South Oman with Alkaline pH

**DOI:** 10.1155/2018/9230143

**Published:** 2018-03-21

**Authors:** Biji Shibulal, Saif N. Al-Bahry, Yahya M. Al-Wahaibi, Abdulkadir E. Elshafie, Ali S. Al-Bemani, Sanket J. Joshi

**Affiliations:** ^1^Department of Biology, College of Science, Sultan Qaboos University, Muscat, Oman; ^2^Department of Petroleum and Chemical Engineering, College of Engineering, Sultan Qaboos University, Muscat, Oman; ^3^Central Analytical and Applied Research Unit, College of Science, Sultan Qaboos University, Muscat, Oman

## Abstract

The identification of potential hydrocarbon utilizing bacteria is an essential requirement in microbial enhanced oil recovery (MEOR). Molecular approaches like proteomic and genomic characterization of the isolates are replacing the traditional method of identification with systemic classification. Genotypic profiling of the isolates includes fingerprint or pattern-based technique and sequence-based technique. Understanding community structure and dynamics is essential for studying diversity profiles and is challenging in the case of microbial analysis. The present study aims to understand the bacterial community composition from different heavy oil contaminated soil samples collected from geographically related oil well areas in Oman and to identify spore-forming hydrocarbon utilizing cultivable bacteria. V4 region of 16S rDNA gene was the target for Ion PGM™. A total of 825081 raw sequences were obtained from Ion torrent from all the 10 soil samples. The species richness and evenness were found to be moderate in all the samples with four main phyla, Firmicutes, Bacteroidetes, Proteobacteria, and Actinobacteria, the most abundant being Firmicutes.* Bacillus *sp. ubiquitously dominated in all samples followed by* Paenibacillus*, which was followed by* Brevibacillus*,* Planococcus*, and* Flavobacterium*. Principal Coordinate Analysis (PCoA) and UPGMA dendrogram clustered the 10 soil samples into four main groups. Weighted UniFrac significance test determined that there was significant difference in the communities present in soil samples examined. It can be concluded that the microbial community was different in all the 10 soil samples with* Bacillus* and* Paenibacillus *sp. as predominating genus. The 16S rDNA sequencing of cultivable spore-forming bacteria identified the hydrocarbon utilizing bacteria as* Bacillus* and* Paenibacillus* sp. and the nucleotide sequences were submitted to NCBI GenBank under accession numbers KP119097–KP119115.* Bacillus* and* Paenibacillus* sp., which were relatively abundant in the oil fields, can be recommended to be chosen as candidates for hydrocarbon utilization study.

## 1. Introduction

Oil production has been experiencing decline in many parts of the world due to oilfield's maturity and an example of such includes the major oilfields in the North Sea [1]. Another major concern is the increasing energy demands due to global population growth and the difficulty in discovering new oilfields as an alternative to the exploited oil fields. Therefore, there is an urge to find out alternative technologies to increase oil recovery from existing oilfields around the world. It is a fact that fossil fuels will still remain the key source of energy, regardless of the gross investments in other energy sources such as biofuels, solar energy, and wind energy. This fact is highlighted by the current global energy production from fossil fuels which currently stand at about 80–90% with oil and gas representing about 60% [2]. During oil production, primary oil recovery can account for 30–40% oil productions, while additional 15–25% can be recovered by secondary methods such as water injection leaving behind about 35–55% of oil as residual oil in the reservoirs [3]. This residual oil is usually the target of many enhanced oil recovery technologies and it amounts to about 2–4 trillion barrels [4] or about 67% of the total oil reserves [5]. Residual oil recovery is at present necessitated for many oil companies and so there is a constant hunt for a cheap and efficient technology which will raise the global oil production as well as the productive life of many oilfields. The recovery of this residual oil is accomplished by enhanced oil recovery (EOR) or tertiary recovery methods which are used in oil industry to increase the production of crude oil. Indigenous or in situ microbial enhanced oil recovery (IMEOR) is one of the techniques in which the inhabitant microbes in the oil reservoirs were stimulated to enhance oil recovery [6]. IMEOR is reported as a cost-effective method [7]. The microbial community composition is influenced by oil reservoir geological conditions and other external factors like nutrient and water flooding, biosurfactant and biopolymer application, and steam injection [8].

For the advancement of a reliable MEOR protocol, understanding the microflora that exist in the oil reservoir is essential. Conventional culture-based techniques have been widely used for bacterial identification and enumeration in oil fields. Culture-based technique is selective and aids in the identification of only a few of the microbes. The problem is even worsened in extreme environmental conditions such as oil fields, where only microbes that can withstand strict circumstances will survive. Therefore, genomic analysis has become an important tool for understanding ecological biodiversity. It evades the need for laboratory cultivation and isolation of individual isolates.

Complex environmental samples need some technique which can read multiple sequences in parallel. Next-Generation Sequencing (NGS) can obtain DNA sequences directly from environmental samples [9, 10]. In the study, the distribution of bacteria in steam-injected heavy oil wells (South Oman) with alkaline pH was characterized using high-throughput sequencing technique, Ion Torrent-Personal Genome Machine (PGM). The spore-forming bacteria in the samples were also identified using culture-based method.

## 2. Materials and Methods

### 2.1. Sample Collection and Preparation

The heavy crude oil contaminated subsurface soil was collected from different heavy oil wells in Southern Oman as described previously [11]. Total ten soil samples were collected aseptically in sterile sampling bags and stored at 4°C for further studies. The samples were kindly provided by a local oil company.

### 2.2. Genomic DNA Isolation

The genomic DNA (gDNA) was isolated from cultivable spore-forming bacteria by boiling suspended soil samples in 10 ml distilled water at 90°C for 30 min to kill all the vegetative cells and enrichment of the bacteria in Bushnell-Haas media (BH media) containing 1% heavy crude oil. The flasks inoculated with the boiled sample supernatant were incubated at 40°C for 24 h and plated on fresh BH agar plates to obtain pure cultures. The gDNA from the isolates and the soil samples was isolated using PowerSoil® DNA Isolation Kit (Mo Bio Laboratories Inc.). The nucleic acid concentration and purity were measured by Thermo Fischer NanoDrop™ 2000/2000 c spectrophotometer.

### 2.3. Identification of Cultivable Spore-Forming Isolates Using 16S rDNA Sequencing

Cultivable spore-forming bacterial isolates were identified by 16S rDNA sequencing using 27F and 1492R universal primers as described previously [11], where briefly the genomic DNA was extracted using PowerSoil DNA Isolation Kit (Mo Bio Laboratories Inc.), and the amplification was performed using T100™ thermal cycler (Bio-Rad, USA). The PCR products were purified using QIAquick PCR purification kit (QIAGen). The BigDye® Terminator v3.1 Cycle Sequencing Kit (Applied Biosystems™) was used for de novo sequencing. The sequencing was done using 3130 XL Genetic Analyzer (Applied Biosystem, Hitachi) at Central Analytical and Applied Research Unit (CAARU), Sultan Qaboos University, and was submitted to NCBI GenBank USA. The dendrogram was constructed using maximum likelihood (ML) methods, respectively, using PHYLIP, the Phylogeny Inference Package program. The ML program uses a Hidden Markov Model (HMM) method of inferring different rates of evolution at different sites [12].

### 2.4. NGS Analysis of Microbial Community in Soil Samples

The metagenomics analysis of all the 10 soil samples was done following the instruction manual of the Ion PGM System. The total gDNA extracted from the soil samples was amplified using 16S Primer Sets targeting hypervariable V4 region: 515F (5′-GTGCCAGCMGCCGCGGTAA-3′) and 806R (5′-GGACTACVSGGGTATCTAAT-3′). The PCR procedure was with an initial denaturation at 95°C for 10 min, 25 cycles of denaturation for 30 sec at 95°C, annealing at 58°C, extension at 72°C for 20 sec, followed by holding at 72°C for 7 min and a final hold at 4°C for ∞. The amplified product was purified using Agencourt® AMPure® XP Reagent and 70% ethanol. The DNA input for library preparation was calculated using Agilent® 2100 Bioanalyzer® and the library was prepared using Ion Plus Fragment Library Kit, following the user instructions. The pooled short amplicons were end-repaired using 5x End Repair Buffer and End Repair Enzyme and purified using Agencourt AMPure XP Reagent and 70% ethanol. Barcoded libraries were prepared using Ion Xpress™ Barcode Adapters 1–16 Kit. The adapters are ligated using DNA Ligase and the nicks were repaired using Nick Repair Polymerase. The DNA template for Ion PGM System was prepared using the Ion PGM Template OT2 400 Kit and the Ion OneTouch™ 2 System following the instructions in Ion PGM Template OT2 400 Kit User Guide. And the library was sequenced using the Ion Personal Genome Machine® (PGM™) System and the Ion PGM Sequencing 400 Kit.

### 2.5. Data Analysis

Primary data analysis was performed with Torrent Suite™ Software v4.0 with automated secondary analysis using Ion Reporter™ Software v4.0. Further analysis was done using a variety of computer packages including Past3, XLstat, NCSS 2007,  “R” and NCSS 2010. Alpha diversity analysis was conducted using QIIME pipeline (version 1.8.0) (QIIME, 2016). Significance reported for any analysis is defined as *p* < 0.05.

Short sequences < 200 bp were removed after depleting primers and barcodes; sequences with ambiguous base calls were also removed. Sequences with homopolymer runs exceeding 6 bp were removed, as the threshold used was 6 bp. Sequences were then denoised and chimeras were removed. Operational taxonomic units (OTUs) were formed at 97% similarity (3% divergence) using UCLUST after removing singleton sequences [13]. RDP classifier version 2.2 was used for taxonomic assignments within the GreenGenes taxonomy [14, 15]. The OTUs were rarefied (randomly subsampled) to 30000 sequences and the signified sequences were aligned to* 16S *reference sequences with PyNAST [16]. The aligned multiple sequences were used to create tree in FastTree [17].

Measures of *α*-diversity, the diversity within the community such as (a) Shannon-Wiener index, also termed the Shannon-Wiener index (*H*′), which evaluates the relative abundance and refers to species richness and species evenness [18] and Simpson index (*D*) [19] and (b) rarefaction, a method for species estimation based on the presence-absence data [20] and (c) Chao 1 [21] plots, based on the number of rare OTUs found in sample were measured using QIIME pipeline [22].

Shannon index is *H*′ = ∑⁡*p*_*i*_ln⁡*p*_*i*_, where *p*_*i*_ is the proportion of individuals found in species *i*, *p*_*i*_ = *n*_*i*_/*N*, where *n*_*i*_ is the number of individuals in species *i* and *N* is the total number of individuals in the community.

Simpson*ʼ*s index = 1 − *D*, where *D* is the dominance.

*D* = ∑*p*_*i*_^2^, where *p*_*i*_ is the proportion of individuals found in species *i*.

SHE analysis was done which is based on the mathematical approach that diversity (Shannon diversity, *H*) is related directly to species richness (*S*) and evenness (*E*) of the distribution [23].

*H* = ln⁡(*S*)  +  ln⁡(*E*), where *H* is Shannon index; *S* is species richness; and *E* is evenness of distribution.

From all OTUs, representative sequences were selected and using QIIME and RDP classifier, taxonomy was assigned for them. Only those phyla which appeared in 75% of the samples were selected. The relative abundance of the genera present in all 10 soil samples was plotted by a heatmap and graphically represented by interactive Krona charts.

*β*-Diversity or community similarity analysis was done by determining the pairwise distances using the phylogenetic distance metric, UniFrac generated by QIIME [24]. Monte Carlo simulations (weighted UniFrac significance test) were done to test the significant difference in OTUs in the samples in the Unweighted Pair Group Method with Arithmetic Mean (UPGMA) tree generated by UniFrac analysis [25], followed by Bonferroni correction [26] to reduce type 1 error rate.

## 3. Results

### 3.1. Sample Collection and Preparation

The soil moisture was determined to be 0.03–0.08 m^3^/m^3^, measured using EM50 Digital data logger, and pH was measured by Jenway 3505 pH meter as 8.5. The viscosity of the heavy crude oil from the well head, which contaminated the soil, was estimated as 4.57°API using RheolabQC Rotation Viscometer. The eTPH of the soil samples ranged within 3.2–4.8%.

### 3.2. Genomic DNA Isolation and Identification of Cultivable Spore-Forming Isolates Using 16S rDNA Sequencing

**Figure 1:**
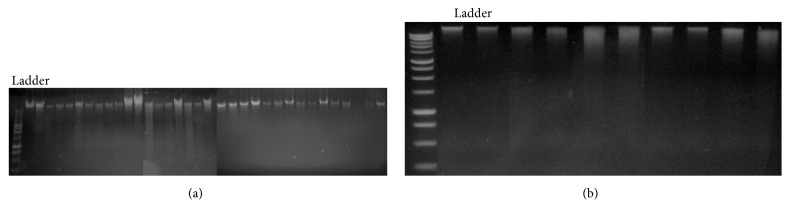
(a) Purified gDNA from isolates electrophoresed on a 1.5% agarose gel and stained with ethidium bromide. First lane contains the 10000 bp molecular weight markers from 300 bp to 10200 bp in length. Other lanes are gDNA samples extracted from the isolates using PowerSoil DNA Isolation Kit; (b) DNA extracted from 10 heavy crude soil samples in 2% agarose gel. Lane 1 is the 1 Kb DNA ladder. 1–10 represent the gDNA from 10 soil samples.

**Figure 2:**
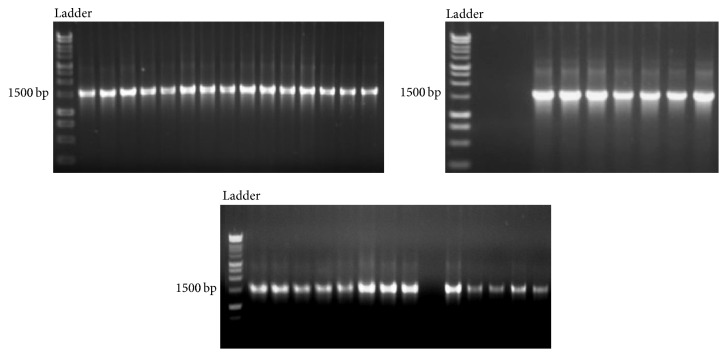
16S rDNA amplified product electrophoresed on a 2% agarose gel and stained with ethidium bromide. First lane contains the 10000 bp molecular weight markers from 300 bp to 10200 bp in length. Other lanes are PCR products amplified using 27F and 1492R primers.

The gDNA from all the isolates (Figure 1(a)) and the soil samples (Figure 1(b)) was extracted using PowerSoil DNA Isolation Kit and was successfully amplified using universal primers for bacteria, 27F and 1492R. The amplified product was 1400 bp (Figure 2).

The purified PCR product was sequenced using 3130 XL Genetic Analyzer and the sequences were submitted to NCBI under the accession numbers KP119097–KP119115 (Table 1).

**Figure 3:**
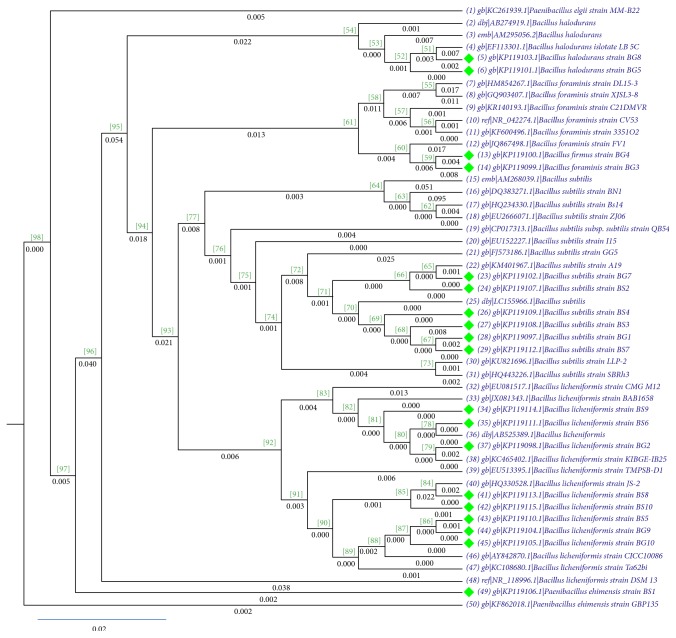
Dendrogram of the isolates using maximum likelihood method (the green dots represent the isolates). The length of each tree segment in the dendrogram represents in units of expected nucleotide substitutions per sites.

The sequences were aligned with the closely related species sequences from NCBI nucleotide blast. The branches with bootstrap values above 70% are reliable. The dendrogram was made using ML (maximum likelihood) method, which assumes every single site of the multiple sequence alignment independently with bootstrapping of 1000 replications. The ln likelihood for the cladogram was −6866.09722. The closer the value of likelihood to zero, the better is the dendrogram (Figure 3). Among the listed isolates in Table 1,* Paenibacillus ehimensis* BS1 showed very good potential for applications in MEOR and heavy crude oil biodegradation [11].

### 3.3. NGS Analysis of Microbial Community in Soil Samples

The gDNA was amplified using specific primers targeting the V4 region of the 16S rRNA bacterial gene using 515F (5′-GTGCCAGCMGCCGCGGTAA-3′) and 806R (5′-GGACTACVSGGGTATCTAAT-3′) for Ion Torrent PGM analysis. A total of 825081 raw sequences were obtained from Ion torrent from all the 10 soil samples. After quality filtering 758305 reads were obtained. Clustering with UCLUST provided 755423 OTUs. The average reads per sample was 75542. The OTUs were rarefied to 30000 sequences and jackknifed at 25000 sequences.

The *α*-diversity measures of the samples were done in QIIME pipeline and Past3. Shannon index (*H*′) and Simpson index (*D*) were calculated for all the samples (Table 2), which revealed that the species richness and relative abundance were present in all the samples, soil sample 2 being with the least diversity.

**Figure 4:**
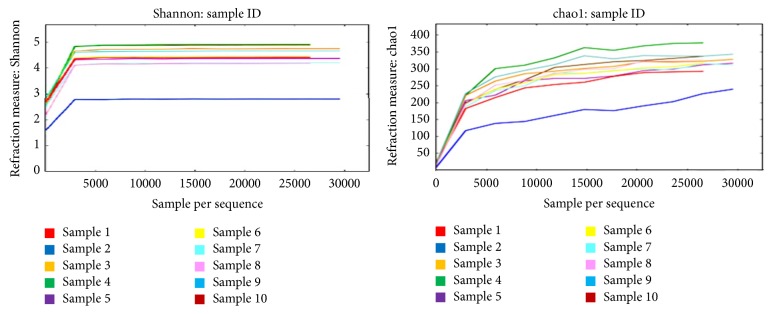
Rarefaction curves showing Shannon diversity index and Chao1 at various sequencing depths. The curve reached a plateau at approximately 5000 sequences.

Rarefaction curves with chao1 and Shannon diversity were plotted with the average number of OTUs at each interval against the size of the subsample [27] (Figure 4). It was found that, for all the 10 soil samples, the curve reached a plateau at approximately 5000 sequences indicating that sequencing depth was sufficient to capture the full scope of microbial diversity.

**Figure 5:**
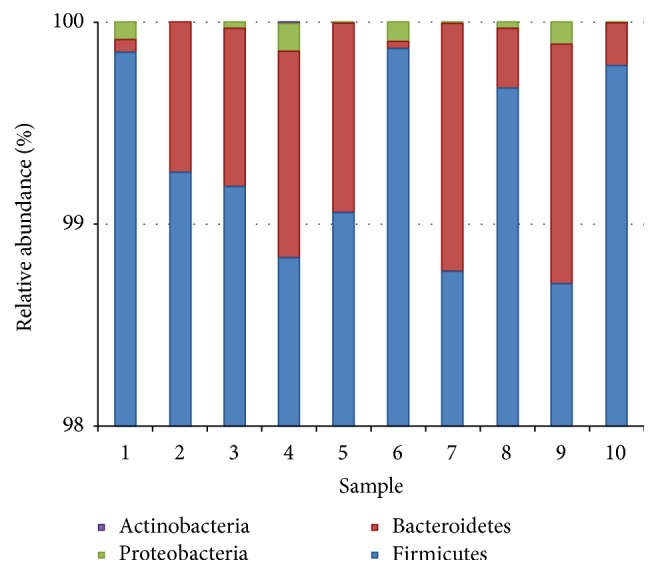
Relative abundance (%) of bacteria phyla. Firmicutes were found to be the most abundant phyla in all the soil samples.

**Figure 6:**
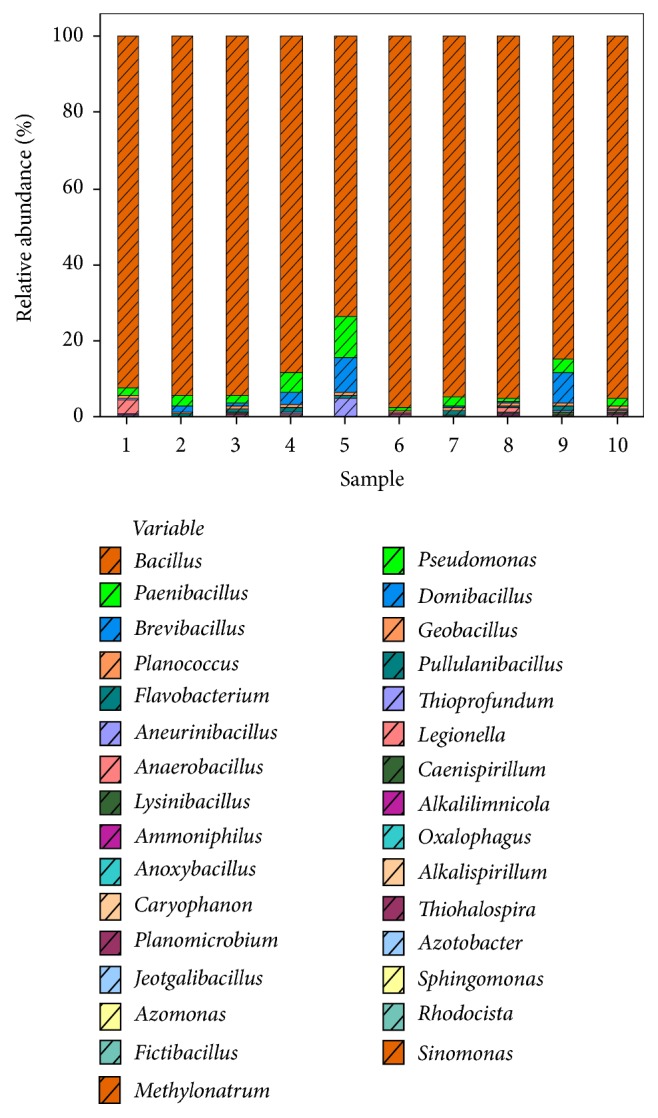
Relative abundance (%) of bacterial genera.* Bacillus* was the most abundant genus followed by* Paenibacillus* and* Brevibacillus*.

Taxonomic comparison of the OTUs identified four main phyla, Firmicutes, Bacteroidetes, Proteobacteria, and Actinobacteria, the most abundant being Firmicutes followed by Bacteroidetes (Figure 5). Further comparison of genus belonging to these phyla showed that* Bacillus* was the most abundant genus followed by* Paenibacillus*, which was followed by* Brevibacillus*,* Planococcus*,* Flavobacterium*, and so forth (Figure 6).

**Figure 7:**
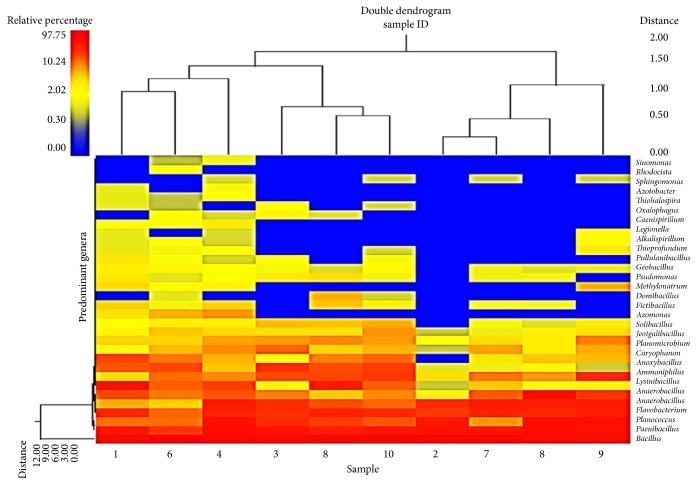
Dual hierarchal dendrogram of the taxonomic classification data, with each sample clustered on the *x*-axis. The relative abundance of* Bacillus*,* Paenibacillus*, and* Brevibacillus* was highest in all the 10 soil samples and the soil samples with more similar microbial populations being mathematically clustered closer together.

The result was confirmed by heatmap analysis in which the highest relative abundance of* Bacillus*,* Paenibacillus*, and* Brevibacillus* was found in all the 10 soil samples and the soil samples with more similar microbial populations were mathematically clustered closer together. The genera (consortium) were used for clustering. Thus, the samples with more similar consortium of genera cluster closer together with the length of connecting lines (top of heatmap) (Figure 7) related to the similarity; shorter lines between two samples indicate closely matched microbial consortium. The heatmap represents the relative percentages of each genus. The predominant genera are represented along the right *y*-axis.

**Figure 8:**
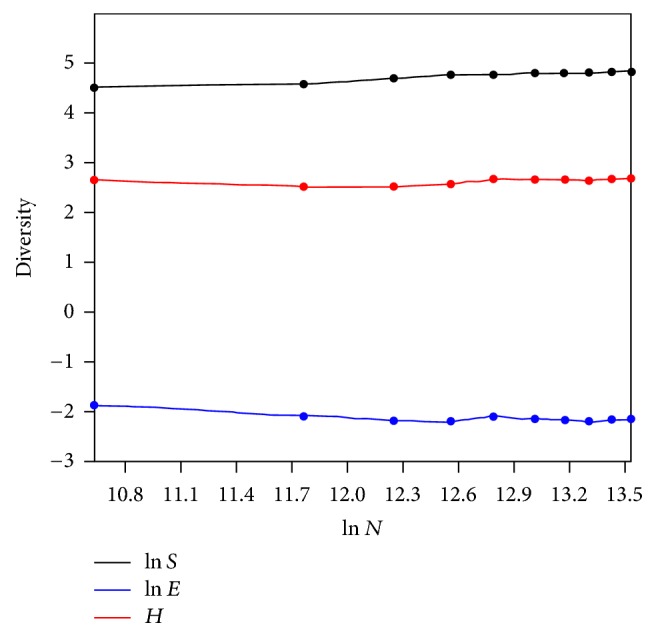
SHE analysis of soil samples showing constant proportion of species in all 10 samples.

SHE analysis was performed to evaluate whether species proportion was similar in all of the 10 soil samples to assess the microbial diversity. The community structure was determined as a log-normal distribution, that is, a few species with high or low abundance and many with intermediate abundance [28] (Figure 8).

**Figure 9:**
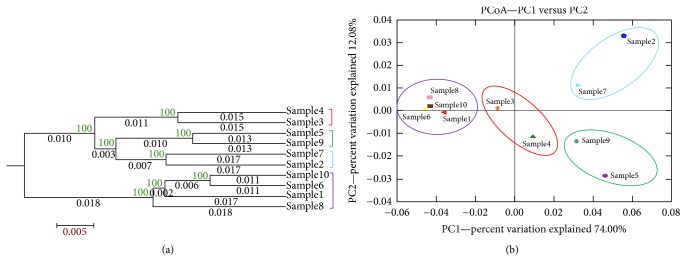
Weighted UniFrac generated (a) Jackknifed UPGMA clustering of soil samples based on the observed OTUs (b) 2D PCoA.

The resemblance between the bacterial communities, *β*-diversity, was measured using UniFrac analysis, which provided a tree-based (Figure 9(a)) Principal Coordinate Analysis (PCoA) graph (Figure 9(b)), which grouped the soil samples to 4 main clusters. The eigenvalues for PC1, PC2, and PC3 were 0.014, 0.002, and 0.001, respectively, and accounted for 74%, 12%, and 6% of the total variance. Both the two approaches revealed the same pattern.

UniFrac *p* values were based on comparisons to 1,000 randomized trees. The *p* values are significant only if they were <0.05 (Table 3).

## 4. Discussion

Ion PGM™ was used to delineate the bacterial community structure of 10 soil samples contaminated with heavy crude oil collected from near oil wells used in the study. The hypervariable V4 region of 16S rDNA sequence was the target region. Stringent quality sequence curation of a total of 825081 raw reads obtained resulted in 84% reduction of initial reads. There are reports stating 50–80% filtering of initial reads [29–31].

The relative abundance of each phyla varied among the 10 soil samples, the predominant phyla observed to be Firmicutes, Bacteroidetes, Proteobacteria, and Actinobacteria, the most abundant being Firmicutes followed by Bacteroidetes. The possible reason for abundance of Firmicutes (*Bacillus *sp.) could be their ability to form endospores to resist adverse conditions of the oil fields, as in desert habitats of Oman [32–35]. Also, because of their metabolic and physiologic adaptability and ability to produce enzyme inhibitors and antibiotics, Firmicutes are considered to be better competitors in natural environment [36]. The moisture content of the soil samples was too low, about 0.03–0.08 m^3^/m^3^, which can be another possible reason. The heatmap analysis of genera showed that* Bacillus* had the highest relative abundance followed by* Paenibacillus* and* Brevibacillus* in all the 10 soil samples.

Measures of *α*-diversity such as Shannon index and Simpson index showed that there was diversity of species OTUs within the community in all the 10 soil samples. Multiple rarefaction curves assembled from each sample's Shannon diversity index reached a plateau at approximately 5000 sequences suggesting that the sequencing depth was sufficient to capture the full scope of microbial diversity [37]. SHE analysis revealed the constant proportion of species in all 10 samples.

*β*-Diversity measured using UniFrac analysis provided a tree-based PCoA (Principal Coordinate Analysis) graph. The two approaches revealed the same pattern of clustering; the Monte Carlo simulation test with Bonferroni corrections revealed that there was significant difference in the communities present in the soil samples examined [24].

The microbial community in soils is determined by the physicochemical parameters such as moisture content, temperature, salinity, and pH [38, 39]. The extreme temperature conditions along with low moisture content (0.03–0.08 m^3^/m^3^) and a slightly alkaline pH will have an impact on the diversity of bacterial community in the soil. Similar results were reported earlier for Tibetan plateau [40]. The presence of heavy crude oil can be another limiting factor for the microbial community.

The identification of cultivable spore-forming isolates by 16S rDNA sequencing from the soil samples resulted in* Paenibacillus* and* Bacillus* sp. The relative abundance of* Bacillus* sp. in the microbial community in heavy crude oil sludge was reported in Saudi Arabia, Oman, and Nigeria. [41–43].* Bacillus* and* Paenibacillus* sp. which were relatively abundant in the oil fields can be recommended to be chosen as candidates for hydrocarbon utilization study. One of our isolates,* Paenibacillus ehimensis* BS1, showed maximum growth in presence of heavy oil and biotransformed it to lighter aliphatic and aromatic compounds demonstrating its potential in EOR and environmental bioremediation under aerobic and anaerobic reservoir conditions [11].

## Figures and Tables

**Table 1:** NCBI GenBank accession numbers for the identified isolates.

Accession No.	Identification
KP119097	*Bacillus subtilis *strain BG1
KP119098	*Bacillus licheniformis *strain BG2
KP119099	*Bacillus foraminis *strain BG3
KP119100	*Bacillus firmus *strain BG4
KP119101	*Bacillus halodurans *strain BG5
KP119102	*Bacillus subtilis *strain BG7
KP119103	*Bacillus halodurans *strain BG8
KP119104	*Bacillus licheniformis *strain BG9
KP119105	*Bacillus licheniformis *strain BG10
KP119106	*Paenibacillus ehimensis *strain BS1
KP119107	*Bacillus subtilis *strain BS2
KP119108	*Bacillus subtilis *strain BS3
KP119109	*Bacillus subtilis* strain BS4
KP119110	*Bacillus licheniformis *strain BS5
KP119111	*Bacillus licheniformis *strain BS6
KP119112	*Bacillus subtilis *strain BS7
KP119113	*Bacillus licheniformis *strain BS8
KP119114	*Bacillus licheniformis *strain BS9
KP119115	*Bacillus licheniformis* strain BS10

**Table 2:** Shannon index and Simpson index.

Samples	Shannon index (*H*′)	Simpson index (*D*)
(1)	2.67	0.89
(2)	1.81	0.73
(3)	2.26	0.81
(4)	2.61	0.86
(5)	2.69	0.90
(6)	2.08	0.79
(7)	2.42	0.85
(8)	2.03	0.71
(9)	2.78	0.90
(10)	2.37	0.84

**Table 3:** Weighted UniFrac significance test with Bonferroni correction.

Sample	1	2	3	4	5	6	7	8	9	10
(1)										
(2)	0.14									
(3)	1	0.01^*∗*^								
(4)	0.87	0.00^*∗*^	1							
(5)	1	0.22	1	0.95						
(6)	1	0.00^*∗*^	1	1	1					
(7)	1	0.75	1	0.4	1	1				
(8)	1	0.00^*∗*^	1	1	1	1	1			
(9)	1	0.00^*∗*^	1	1	1	1	0.63	1		
(10)	1	0.00^*∗*^	1	1	1	1	1	1	1	

^*∗*^ *p* < 0.05: significant.

## References

[1] Aleklett K., Höök M., Jakobsson K., Lardelli M., Snowden S., Söderbergh B. (2010). The Peak of the Oil Age - Analyzing the world oil production Reference Scenario in World Energy Outlook 2008. *Energy Policy*.

[2] Graus W., Roglieri M., Jaworski P., Alberio L., Worrell E. (2011). The promise of carbon capture and storage: Evaluating the capture-readiness of new EU fossil fuel power plants. *Climate Policy*.

[3] Cosse R., Koch M. (1993). Basics of reservoir engineering. *Pure and Applied Geophys*.

[4] Hall C., Tharakan P., Hallock J., Cleveland C., Jefferson M. (2003). Hydrocarbons and the evolution of human culture. *Nature*.

[5] Bryant R. S., Stepp A. K., Bertus K. M., Burchfield T. E., Dennis M. (1993). Microbial-Enhanced Waterflooding Field Pilots. *Developments in Petroleum Science*.

[6] Sunde E., Beeder J., Nilsen R., Torsvik T. (1992). Aerobic microbial enhanced oil recovery for offshore use. *SPE/DOE Enhanced Oil Recovery Symposium*.

[7] Geetha S. J., Banat I. M., Joshi S. J. (2018). Biosurfactants: Production and potential applications in microbial enhanced oil recovery (MEOR). *Biocatalysis and Agricultural Biotechnology*.

[8] Magot M., Ollivier B., Magot M. (2005). Indigenous microbial communities in oil fields. *Petroleum Microbiology*.

[9] Schuster S. C. (2008). Next-generation sequencing transforms today's biology. *Nature Methods*.

[10] Sogin M. L., Morrison H. G., Huber J. A. (2006). Microbial diversity in the deep sea and the underexplored ‘rare biosphere’. *Proceedings of the National Acadamy of Sciences of the United States of America*.

[11] Shibulal B., Al-Bahry S. N., Al-Wahaibi Y. M., Elshafie A. E., Al-Bemani A. S., Joshi S. J. (2017). The potential of indigenous Paenibacillus ehimensis BS1 for recovering heavy crude oil by biotransformation to light fractions. *PLoS ONE*.

[12] Felsenstein J., Churchill G. A. (1996). A Hidden Markov Model approach to variation among sites in rate of evolution. *Molecular Biology and Evolution*.

[13] Edgar R. C. (2010). Search and clustering orders of magnitude faster than BLAST. *Bioinformatics*.

[14] Liu Z., Desantis T. Z., Andersen G. L., Knight R. (2008). Accurate taxonomy assignments from 16S rRNA sequences produced by highly parallel pyrosequencers. *Nucleic Acids Research*.

[15] Schloss P. D., Westcott S. L. (2011). Assessing and improving methods used in operational taxonomic unit-based approaches for 16S rRNA gene sequence analysis. *Applied and Environmental Microbiology*.

[16] Caporaso J. G., Bittinger K., Bushman F. D., DeSantis T. Z., Andersen G. L., Knight R. (2010). PyNAST: a flexible tool for aligning sequences to a template alignment. *Bioinformatics*.

[17] Price M. N., Dehal P. S., Arkin A. P. (2010). FastTree 2—approximately maximum-likelihood trees for large alignments. *PLoS ONE*.

[18] Shannon C. E. (1949). Communication theory of secrecy systems. *Bell Labs Technical Journal*.

[19] Simpson E. H. (1949). Measurement of diversity. *Nature*.

[20] Simberloff D. (1972). Properties of the Rarefaction Diversity Measurement. *The American Naturalist*.

[21] Chao A. (1984). Nonparametric estimation of the number of classes in a population. *Scandinavian Journal of Statistics*.

[22] Meyer F., Paarmann D., D'Souza M. (2008). The metagenomics RAST server—a public resource for the automatic phylogenetic and functional analysis of metagenomes. *BMC Bioinformatics*.

[23] Hayek L. C., Buzas M. A. (1997). *Surveying Natural Populations: Quantitative Tools for Assessing Biodiversity*.

[24] Lozupone C., Knight R. (2005). UniFrac: a new phylogenetic method for comparing microbial communities. *Applied and Environmental Microbiology*.

[25] Lozupone C. A., Knight R. (2007). Global patterns in bacterial diversity. *Proceedings of the National Acadamy of Sciences of the United States of America*.

[26] Tenesa A., Farrington S. M., Prendergast J. G. D. (2008). Genome-wide association scan identifies a colorectal cancer susceptibility locus on 11q23 and replicates risk loci at 8q24 and 18q21. *Nature Genetics*.

[27] Gotelli N. J., Colwell R. K. (2001). Quantifying biodiversity: procedures and pitfalls in the measurement and comparison of species richness. *Ecology Letters*.

[28] Buzas M. A., Hayek L.-A. C. (2005). On richness and evenness within and between communities. *Paleobiology*.

[29] Gloor G. B., Hummelen R., Macklaim J. M. (2010). Microbiome profiling by illumina sequencing of combinatorial sequence-tagged PCR products. *PLoS ONE*.

[30] Indugu N., Bittinger K., Kumar S., Vecchiarelli B., Pitta D. (2016). A comparison of rumen microbial profiles in dairy cows as retrieved by 454 Roche and Ion Torrent (PGM) sequencing platforms. *PeerJ 4*.

[31] Caporaso J. G., Lauber C. L., Walters W. A. (2011). Global patterns of 16S rRNA diversity at a depth of millions of sequences per sample. *Proceedings of the National Acadamy of Sciences of the United States of America*.

[32] Vreeland R. H., Rosenzweig W. D., Powers D. W. (2000). Isolation of a 250 million-year-old halotolerant bacterium from a primary salt crystal. *Nature*.

[33] Jang L. K., Chang P. W., Findley J. E., Yen T. F. (1983). Selection of bacteria with favorable transport properties through porous rock for the application of microbial-enhanced oil recovery. *Applied and Environmental Microbiology*.

[34] Clark J., Munnecke D., Jenneman G. (1981). Insitu microbial enhancement of oil production. *Developments in Industrial Microbiology*.

[35] McInerney M. J., Menzie D. E., Jenneman G. E. (1983). *The Use of Microorganisms in Enhanced Oil Recovery*.

[36] Pandey S., Sree A., Dash S. S., Sethi D. P., Chowdhury L. (2013). Diversity of marine bacteria producing beta-glucosidase inhibitors. *Microbial Cell Factories*.

[37] Aravindraja C., Viszwapriya D., Karutha Pandian S. (2013). Ultradeep 16S rRNA sequencing analysis of geographically similar but diverse unexplored marine samples reveal varied bacterial community composition. *PLoS ONE*.

[38] Wang L.-Y., Duan R.-Y., Liu J.-F., Yang S.-Z., Yang J.-D., Mu B.-Z. (2012). Molecular analysis of the microbial community structures in water-flooding petroleum reservoirs with different temperatures. *Biogeosciences*.

[39] Xiao M., Sun S.-S., Zhang Z.-Z. (2016). Analysis of bacterial diversity in two oil blocks from two low-permeability reservoirs with high salinities. *Scientific Reports*.

[40] Zhang X. F., Zhao L., Xu S. J., Liu Y. Z., Liu H. Y., Cheng G. D. (2013). Soil moisture effect on bacterial and fungal community in Beilu River (Tibetan Plateau) permafrost soils with different vegetation types. *Journal of Applied Microbiology*.

[41] Albokari M., Mashhour I., Alshehri M., Boothman C., Al-Enezi M. (2015). Characterization of microbial communities in heavy crude oil from Saudi Arabia. *Annals of Microbiology*.

[42] Allamin I., Ijah U., Ismail H., Riskuwa M. (2014). Occurrence of hydrocarbon degrading bacteria in soil in Kukawa, Borno State. *International Journal of Environment*.

[43] Al-Sayegh A., Al-Wahaibi Y., Al-Bahry S., Elshafie A., Al-Bemani A., Joshi S. (2015). Microbial enhanced heavy crude oil recovery through biodegradation using bacterial isolates from an Omani oil field. *Microbial Cell Factories*.

